# Kernel Principal Component Analysis for dimensionality reduction in fMRI-based diagnosis of ADHD

**DOI:** 10.3389/fnsys.2012.00074

**Published:** 2012-11-09

**Authors:** Gagan S. Sidhu, Nasimeh Asgarian, Russell Greiner, Matthew R. G. Brown

**Affiliations:** ^1^Department of Computing Science, University of AlbertaEdmonton, AB, Canada; ^2^Alberta Innovates Center for Machine Learning, University of AlbertaEdmonton, AB, Canada; ^3^General Analytics Inc.Edmonton, AB, Canada^†^; ^4^Department of Psychiatry, University of AlbertaEdmonton, AB, Canada

**Keywords:** ADHD, classifier, diagnosis, FFT, fMRI, kernel methods, machine learning, PCA

## Abstract

This study explored various feature extraction methods for use in automated diagnosis of Attention-Deficit Hyperactivity Disorder (ADHD) from functional Magnetic Resonance Image (fMRI) data. Each participant's data consisted of a resting state fMRI scan as well as phenotypic data (age, gender, handedness, IQ, and site of scanning) from the ADHD-200 dataset. We used machine learning techniques to produce support vector machine (SVM) classifiers that attempted to differentiate between (1) all ADHD patients vs. healthy controls and (2) ADHD combined (ADHD-c) type vs. ADHD inattentive (ADHD-i) type vs. controls. In different tests, we used only the phenotypic data, only the imaging data, or else both the phenotypic and imaging data. For feature extraction on fMRI data, we tested the Fast Fourier Transform (FFT), different variants of Principal Component Analysis (PCA), and combinations of FFT and PCA. PCA variants included PCA over time (PCA-*t*), PCA over space and time (PCA-*st*), and kernelized PCA (kPCA-*st*). Baseline chance accuracy was 64.2% produced by guessing healthy control (the majority class) for all participants. Using only phenotypic data produced 72.9% accuracy on two class diagnosis and 66.8% on three class diagnosis. Diagnosis using only imaging data did not perform as well as phenotypic-only approaches. Using both phenotypic and imaging data with combined FFT and kPCA-*st* feature extraction yielded accuracies of 76.0% on two class diagnosis and 68.6% on three class diagnosis—better than phenotypic-only approaches. Our results demonstrate the potential of using FFT and kPCA-*st* with resting-state fMRI data as well as phenotypic data for automated diagnosis of ADHD. These results are encouraging given known challenges of learning ADHD diagnostic classifiers using the ADHD-200 dataset (see Brown et al., 2012).

## 1. Introduction

Over the past decade, many researchers have applied statistical analysis to functional Magnetic Resonance Images (fMRIs) in order to better understand neuropsychiatric phenomena. Much of this research has used fMRI to identify group differences between subjects that have a specific neuropsychiatric disorder and healthy controls (Purdon et al., 2011). There has also been a more recent focus on developing methodologies for diagnosing neuropsychiatric illnesses with high accuracy using advanced statistical methods (Cecchi et al., 2009). With large-scale fMRI studies including hundreds of participants or more, *tractability* becomes an issue. Each fMRI volume contains roughly ~10^5^ voxel locations, each with a waveform that may be composed of hundreds of time points. Some patterns in these voxel waveforms may be diagnostic for a neuropsychiatric disorder, but there is also substantial variance in the data that is not related to diagnosis. The accuracy of computerized diagnosis can be facilitated by feature extraction during preprocessing. Feature extraction methods reduce the size of the original fMRI data by extracting a smaller number of features for each subject (i.e., reducing the dimensionality of each subject's data). The challenge is to do this *without* diminishing the diagnostic value of each subject's data—i.e., while preserving the information needed to produce an effective classifier (Alpaydin, 2004).

We built a system capable of learning a diagnostic classifier. We investigated the effects on diagnostic accuracy of using different fMRI data dimensionality reduction methods within this system. Our learning system consisted of two stages, each of which was comprised of many components. At training time, the first stage used subjects' scans to produce a *diagnostic classifier*. Then at performance time, the second stage used the (machine learner) classifier to produce a diagnosis for a novel subject—i.e., a subject whose data were not used to develop the classifier. Note that the objective of producing a classifier is different from the more standard associative approach. Associative studies investigate differences between groups, such as differences in average fMRI activation levels (in various brain regions) between different diagnostic groups (Eisenberger et al., 2003). Significant differences between group averages can exist in the presence of substantial overlap among the members of the different groups (Brown et al., 2012). In contrast, diagnostic classifiers must consider individual differences—that is patterns in the data that differentiate individuals into different groups, as opposed to differences in group averages.

**Figure 1:**
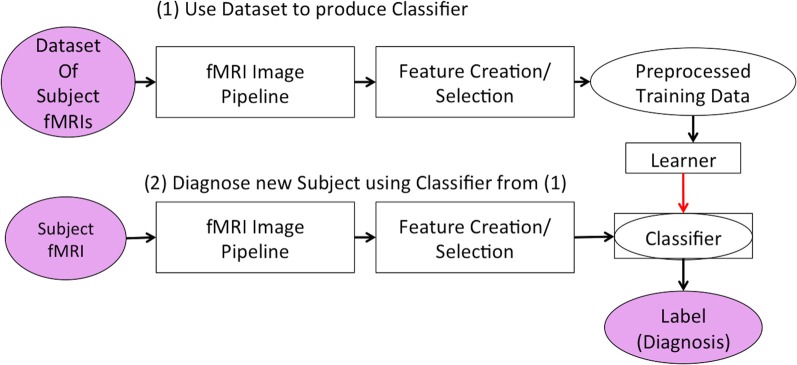
**Our learning system in two stages**. The first stage developed the classifier responsible for diagnosing new subjects. The second stage used the classifier to generate a predicted diagnosis for a participant.

As shown in Figure 1, both stages first ran the “fMRI Image Pipeline” and then reduced the fMRI data's dimensionality in the “Feature Creation/Selection” component. This study focused on this second “Feature Creation/Selection” component. We investigated whether reducing fMRI dimensionality over both the spatial and temporal dimensions (in contrast to reducing only the spatial or only the temporal dimensionality) would improve the diagnostic system's discrimination of Attention-Deficit Hyperactivity Disorder (ADHD) patients from healthy controls.

**Figure 2:**
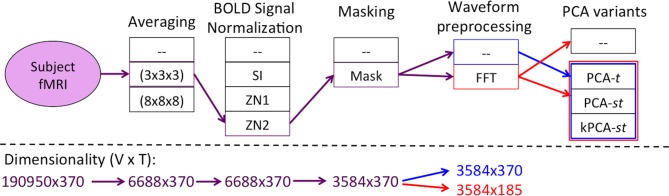
**Flow of the “Feature Creation/Selection” component of our diagnostic system, where the red and blue lines denote the processes with and without FFT as a preprocessing step, respectively**. The PCA variant step involved developing the respective variant's data matrix and processing it by one of PCA-*t* (Figure 4), PCA-*st*, or kPCA-*st* (Figure 5). The fMRI dimensionality is given beneath each step, written as *r* × c, where the number of rows (*r*) and columns (*c*) represent the spatial and temporal dimensions, respectively.

Figure 2 summarizes the “Feature Creation/Selection” step. After several fixed steps (see section 2), we then considered seven dimensionality reduction processes. The first three processes applied a variant of Principal Component Analysis (PCA). These variants applied PCA over the temporal dimension PCA over time (PCA-*t*) or over both the temporal and spatial dimensions [PCA over space and time (PCA-*st*) and the kernelized variant (kPCA-*st*)]. The other four dimensionality reduction process involved applying the Fast Fourier Transform (FFT) to each voxel's waveform and then using the output of the FFT as input either directly to the learner or to one of the three PCA variants.

In this study, we opted for a *biologically naive* approach to fMRI dimensionality reduction. That is, we did not use any prior biological information about the brain, such as the locations of brain regions known to be affected by ADHD. In another study (Brown et al., 2012), we tested feature extraction methods based on group differences between ADHD patients and controls, but these did not provide high diagnostic accuracy. One advantage of such a biologically naive approach is that it was not biased by nor limited to previously-known group differences between patients and controls.

We tested these seven feature extraction and classifier learning processes on the ADHD-200 Global Competition dataset (Fair et al.). Automated diagnosis of ADHD using this dataset has proven to be very challenging for the scientific community in general. Twenty-one teams took part in the ADHD-200 Global Competition. Our team submitted a classifier that used only the phenotypic data to produce a diagnosis. This classifier achieved the best accuracy (62.5%) of any submission (Brown et al., 2012; Alberta Innovates Center for Machine Learning). For comparison, guessing “healthy control” for every participant in the ADHD-200 Competition's holdout dataset produced an accuracy of 55%. See Brown et al. (2012) for further discussion.

The first three dimensionality reduction processes applied a variant of PCA to subject's voxel waveforms. For PCA-*t*, we represented the data from all subjects as a matrix, with each row containing the original waveform from one voxel location from one subject (Figure 4). That is, the number of rows equaled the number of subjects times the number of voxel locations. The voxel configuration was identical in all subjects after spatial normalization of fMRI data, performed during preprocessing as described below. The PCA-*t* approach ran PCA on this large matrix. This PCA-*t* method has various applications in fMRI analysis (Andersen et al., 1999). PCA-*t* compresses each waveform into a smaller number of features (specifically, weightings on principal component vectors). We then trained a classifier using the set of all such compressed waveforms for each subject. For the second PCA variant, PCA-*st*, we first combined all subjects' data into a large matrix in which each row contained the waveforms from all voxels for a given subject (Figure 5). That is, for a given row, which corresponded to a given subject, we concatenated the waveforms from all voxel locations for that subject to construct the row vector. The PCA-*st* method then performed PCA on this matrix. The third PCA variant was kernel Principal Component Analysis (kPCA-*st*), described in section 2.7, which introduced nonlinear compression of the data.

The remaining four dimensionality reduction processes applied the FFT to the waveforms of all voxels of all subjects, then extracted the complex magnitude for each of the FFT frequency components. The simplest FFT-based feature extraction process used these magnitudes as input features for the learner. The other three FFT-based feature extraction procedures used the magnitude values of the FFT frequency components as input to one of the three PCA variants described in the previous paragraph.

We compared diagnostic systems based on feature extraction (dimensionality reduction) methods that differed in terms of (1) reduction over both the spatial and temporal dimensions vs. reduction over only the temporal dimension, (2) linear vs. nonlinear (kernelized) PCA, and (3) initial processing with vs. without FFT.

Section 2 starts with descriptions of the data and our fMRI Preprocessing Pipeline. It then describes the FFT, PCA, and kPCA-*st* methodologies, as well as our testing methodology. Section 3 discusses the results for each of these methods on the dataset provided by the ADHD-200 Competition. Briefly, the best diagnostic accuracy was achieved using a combination of phenotypic data and fMRI data with feature extraction based on FFT followed by kPCA-*st*.

## 2. Materials and methods

This section presents the overall approach, based on Figures 1, 2. Section 2.1 gives details of the data we used. Section 2.2 presents the first step, the fMRI Preprocessing Pipeline. The remaining subsections describe the Feature Creation/Selection methodology, which we summarize in Figure 2. Section 2.3 discusses “Averaging.” Section 2.4 discusses “BOLD Signal Intensity Normalization and Masking.” Section 2.5 addresses waveform processing using FFT. Sections 2.6 and 2.7 present the PCA variants and “Kernel Methods,” including the kernelized PCA variant, kPCA-*st*. Section 2.8 describes our “Testing Methodology.”

### 2.1. Data

Automated ADHD diagnosis protocols were tested on data from the ADHD-200 Global Competition dataset (Fair et al.). We used 668 of the 776 participants from the ADHD-200 Competition's training dataset, after removing the 108 participants whose scans did not pass quality assurance checks by the ADHD-200 data curators. This 668-subject dataset, summarized in Table 1 left, contained the age, gender, handedness, IQ scores, and scanning site, as well as a resting-state fMRI scan for each of 429 healthy controls, 141 ADHD-combined (ADHD-c) patients, and 98 ADHD-inattentive (ADHD-i) patients. Most participants were adolescents, while the remainder were children or young adults. We tested each learned classifier against the ADHD-200 Competition's holdout set (Table 1 right side). We used data from only 171 of the 197 holdout participants, as we excluded the 26 participants from the Brown University site, for whom diagnostic labels were not released.

**Table 1:** **Distribution of ADHD patients and control subjects contained in the original (left) and holdout (right) datasets**.

**Hospital**	**Number of subjects**	**Control group**	**ADHD**		**Hospital**	**Number of subjects**	**Control group**	**ADHD**	
			**combined**	**inattentive**				**combined**	**inattentive**
Kennedy Kriegler	78	58	15	5	Kennedy Kriegler	11	8	3	0
Institute (KKI)					Institute (KKI)				
NeuroIMAGE	38	16	12	0	NeuroIMAGE	25	14	11	0
Peking University	194	116	29	49	Peking University	51	27	10	14
Oregon Health Science	64	36	17	11	Oregon Health Science	34	28	5	1
University (OHSU)					University (OHSU)				
New York	188	91	64	33	New York	41	12	22	7
University (NYU)					University (NYU)				
University	66	66	0	0	University	9	5	0	4
of Pittsburgh					of Pittsburgh				
Washington University	40	40	0	0	Washington University	0	0	0	0

### 2.2. fMRI preprocessing pipeline

The ADHD-200 Global Competition organizers provided both raw and preprocessed data. Instead of using the preprocessed data that they provided, we used our own pipeline to preprocess the raw fMRI images using SPM8 (The FIL Methods Group) and in-house MATLAB code (as specified below). For each subject, the preprocessing pipeline involves:

1. Six parameter rigid body motion correction (SPM8)
2. Co-registration of functional scans to each subject's respective anatomical scan (SPM8)
3. Nonlinear spatial warping of anatomical volume to MNI T1 (Evans et al., 1993) template space at 1 × 1 × 1 mm resolution (SPM8)
4. Warping of the fMRI volumes into T1 template space at 3 × 3 × 3 spatial resolution, using the same warping parameters computed in the previous step without re-estimation.
5. Application of an 8 mm full width at half maximum (FWHM) Gaussian spatial filter to the fMRI volumes (SPM8)
6. Truncation of all resting-state fMRI scanning data to a 185 s duration (as this is the shortest time used in all hospitals), followed by temporal linear interpolation to a sampling rate of 2 Hz.

After this preprocessing, every subject's fMRI data had the same spatiotemporal dimensions (57 × 67 × 50 voxels with waveforms over 370 time points) and sampling parameters (3 × 3 × 3 mm voxels spatially, 0.5 s sampling period).

We did not model the fMRI data with explicit nuisance regressors (e.g., run offsets, low frequency sinusoids, and motion parameters) as this is not standard practice in resting state fMRI analysis. Inclusion of nuisance regressors is common in general linear model (GLM)-based analysis of fMRI when the participant performs a structured task. In contrast, many functional connectivity analyses, including those based on PCA and FFT, are not based on the GLM and have no straightforward way to incorporate nuisance regressors. Instead, we removed known or suspected sources of noise by applying filtering and other corrections to the data prior to the main analysis. We performed standard rigid body motion correction and, where appropriate, time courses were mean-centered. This addressed the issue of differences in mean scan intensity, which is the same issue addressed by run offset predictors in a GLM. Resting-state fMRI analyses typically treat low frequency components—i.e., in the range of 0.001–0.1 Hz—as containing meaningful signal and often focus on only those components (Biswal et al., 1995; Yu-Feng et al., 2007). We therefore did not perform high pass temporal filtering to remove those frequency components as this may have removed diagnostically-useful information from the data.

### 2.3. Averaging

After preprocessing (section 2.2), each subject's fMRI data consisted of volumes with spatial size *L* × *W* × *H* voxels. Each voxel had a waveform of length *T* = 370, meaning that each subject's scan had a total spatiotemporal dimensionality of *L* × *W* × *H* × *T*, which here was 57 × 67 × 50 × 370 real values per scan.

**Figure 3:**
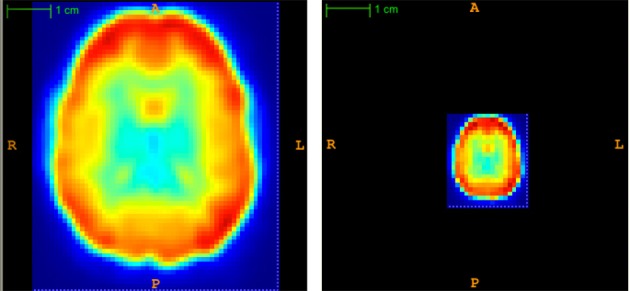
**Temporal view of subject's original (left) and averaged (right) volume at time point *t* = 1**. Averaging reduced the spatial dimensionality by a factor of 3 × 3 × 3 = 27.

Each subject's 57 × 67 × 50 × 370 fMRI scan required roughly 282 MB of memory when represented as a single-precision, four dimensional array. Applying PCA (section 2.6) to the fMRI data from all 668 subjects at once would have required 188.78 GB in memory, which would have strained our computing resources, both directly and indirectly (by thrashing). To address the high dimensionality of the data, we first reduced the spatial dimensionality using “averaging”—i.e., replacing each *k* × k × k sub-volume by its average intensity value, for each time point *t* ∈ [1, …, T]. Partial volumes on the edge of the scan were dropped. This procedure reduced the data size by a factor of ≥ *k*^3^. Figure 3 illustrates a subject's fMRI data before and after averaging. For this study, every subject's fMRI scan was averaged by taking the mean over 3 × 3 × 3 subvolumes (i.e., *k* = 3), resulting in ⌊57/3⌋ × ⌊67/3⌋ × ⌊50/3⌋ = 19 × 22 × 16 = 6688 voxel waveforms. We also tried averaging over 8 × 8 × 8 subvolumes (i.e., *k* = 8) but found that all methods performed poorly on this averaged data.

One potential criticism of using this averaging method is that it discards information by reducing the spatial resolution. However, note that subjects' fMRI scans were already spatially smoothed with an 8 mm FWHM Gaussian filter during preprocessing. We argue that averaging with *k* = 3 did not impose a substantial, additional reduction in spatial resolution. We did perform a small number of tests without averaging and found that excluding the averaging step did not improve diagnostic performance (results not shown), though it did increase the computation time substantially.

### 2.4. Bold signal intensity normalization and masking

After preprocessing and averaging, we used BOLD signal intensity normalization to address between-subject differences in waveform magnitude introduced by differences in scanner configuration or image registration. We considered three different signal normalization methods (also see Desmond and Glover, 2002). Let μ_*x*_*i*__ be the mean intensity over a voxel's waveform *x*_*i*_ and σ_*x*_*i*__ be the standard deviation of the intensity values in *x*_*i*_. Then, for each voxel *i* = 1, …, *V*:

**Percent Signal Change**:

$$x_{i,\;norm}=\frac{(x_{i}-\mu_{x_{i}})\times 100}{\mu_{x_{i}}}$$

**Z-Score Normalization 1**:

$$x_{i,\;norm}=\frac{x_{i}-\mu_{x_{i}}}{\sigma_{x_{i}}}$$

The classification accuracies when using these methods were no better than the baseline, which suggested that normalizing waveforms according to their *local* properties—i.e., the voxel waveform's mean and/or standard deviation—was not useful for discriminating patients from controls.

We also normalized voxel waveform values using the global mean, μ_*x*_, and standard deviation, σ_*x*_, calculated from intensity values over a subject's *entire* fMRI scan:

**Z-Score Normalization 2 (ZN2)**:

$$x_{i,\;norm}=\frac{x_{i}-\mu_{x}}{\sigma_{x}}$$

In initial tests, we found that ZN2 signal normalization resulted in the most accurate diagnostic performances, and we used it rather than the other two signal normalization methods. After performing ZN2 signal normalization, we applied a mask that removed voxels outside of the brain. This left only 3584 voxel waveforms per subject.

### 2.5. Fourier transforms

We used MATLAB's implementation of the FFT (Oppenheim et al., 1999) on each of the 3584 voxels' waveforms. Each such waveform contained 370 time points, and the FFT produced 185 complex-valued Fourier component weights. We extracted the magnitude and discarded the phase for each frequency component. After FFT, each subject's data consisted of 3584 × 185 = 633,040 values (features). As shown in Figure 2, we used these values as input to the next step—either a PCA variant or the learner itself.

### 2.6. Principal component analysis

PCA is a dimensionality reduction technique that computes the linear combination of features—e.g., the intensities of a specific voxel at a single time, over the set of data points—that have high variance (Rencher, 2002). Instead of representing a data point using the original features, we can “project” those original features onto a smaller number of principal components and know that this re-encoding “captures” a large proportion of variance in the data. We assume readers are familiar with PCA and discuss its relevant theoretical properties in Appendices C.1 and C.2.

#### 2.6.1. PCA compression over time: PCA-*t*

Here we describe PCA-*t*, which is a standard approach to reducing fMRI dimensionality. Its purpose is to capture the variance over waveforms by selecting the top *m* components—i.e., the ones that are responsible for the largest proportion of the variance over voxel waveforms. PCA-*t*'s principal components have been interpreted as representing networks of activation (Huettel et al., 2009).

Andersen et al. used primates' fMRI data to show that PCA-*t*'s largest principal components captured the systematic structure—i.e., voxel activation patterns—based on the assumption that the smaller *T* − m principal components were essentially noise. Andersen et al. also argued that a degree of subjectivity is involved in interpreting the projections produced by PCA-*t* (Andersen et al., 1999). Note that our supervised learning framework provides an *objective* way to evaluate various dimensionality reduction techniques, based on the performance “downstream,” of the resulting classifier. We used this supervised learning framework to evaluate whether using PCA-*t* feature extraction would allow us to learn a classifier that could discriminate ADHD patients from controls.

**Figure 4:**
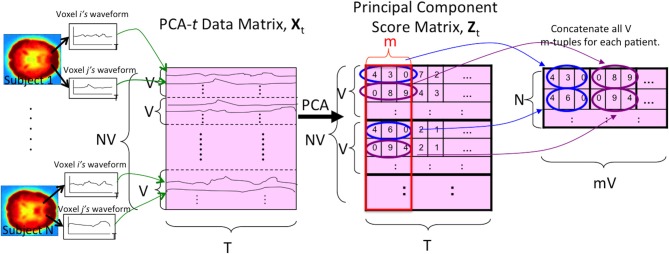
**The PCA-*t* process**. *V* is the number of voxels per subject. *N* is the number of subjects. *T* is the length of each voxel's waveform in number of time points. All *V* voxel waveforms from all *N* subjects were represented by the rows of the data matrix **X**_*t*_. After running PCA on this matrix, the *m* largest principal component scores of each subject's waveforms were used as the reduced-dimension features. Note that the input signal was always the 3 × 3 × 3 averaged (spatially-reduced) version within the masked sub-volume. For some tests (Figure 2), the “waveform” used to form the PCA-*t* matrix was the FFT of the original waveforms (see section 2.5).

PCA-*t* treated the waveform from each voxel from each subject as a data point. As shown in Figure 4, PCA-*t* took as input a data matrix **X**_*t*_ and produced a matrix **Z**_*t*_ whose principal components captured over 99% of the variance in **X**_*t*_ (Equation A.9). Each subject's fMRI data were first averaged, BOLD signal normalized, and masked and then reshaped from a 4D representation to a 2D one (by vectorizing the spatial dimensions). This procedure produced a *V* × T matrix for each subject. All *N* subjects' matrices were concatenated vertically to produce the *NV* × T data matrix **X**_*t*_. **X**_*t*_ was projected onto the low-dimensional eigenvector matrix *E* ∈ ℝ^T × m^ (Equation A.7) to produce the *NV* × m principal component scores matrix **Z**_*t*_, where the *m NV*-dimensional principal components (columns) contained the *N* subjects' principal component scores.

#### 2.6.2. PCA compression over time and space: PCA-*st*

**Figure 5:**
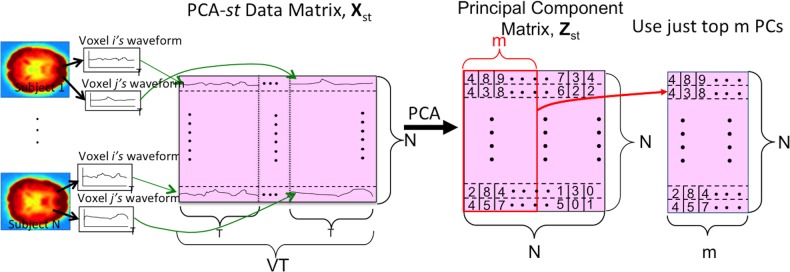
**The PCA-*st* process**. *V* is the number of voxels per subject. *N* is the number of subjects. *T* is the length of each voxel's waveform in number of time points. Each subject's voxel waveforms were horizontally concatenated to form one row of the data matrix **X**_*st*_ used by PCA-*st* and kPCA-*st*. Note that the input signal was always the 3 × 3 × 3 reduced version within the masked sub-volume. For some tests (Figure 2), the “waveform” used to form the PCA-*st* matrix was the FFT of the original waveforms. Note that we can replace the first step, PCA, with kernel-PCA (kPCA) to represent the kPCA-*st* process.

We also tested a variant of PCA, PCA-*st*, which considered both spatial and temporal aspects of the fMRI data. PCA-*st* treated the entire fMRI scan from each subject as a datapoint. In PCA-*st*, we applied PCA to an *N* × *VT* data matrix **X**_*st*_ in which each row represented the fMRI data from one subject. After averaging, BOLD signal normalization, and masking a given subject's fMRI data, all waveforms from all voxels for that subject were concatenated, and the resulting row vector comprised one row of the PCA data matrix **X**_*st*_. Figure 5 shows the data matrix **X**_*st*_, as well as the matrix **Z**_*st*_ produced by PCA. **Z**_*st*_'s principal components captured over 99% of the variance over the waveforms (Equation A.9).

PCA-*st* used the dual trick to recover the high-dimensional eigenvectors of the covariance matrix **S**_*st*_ from the low-dimensional eigenvectors of **K**_*st*_ (Equation A.6). We then projected **X**_*st*_ onto the *VT* × *N* eigenvector matrix (Equation A.8). This produced an *N* × *N* principal component matrix **Z**_*st*_ that contained *N* principal components for every subject. Each principal component was the dot product between the respective subject's *VT*-dimensional vectorized imaging data and one of the *N VT*-dimensional eigenvectors of **S**_*st*_.

(1) $$Z_{st}(j,\;i)=\langle x_{j},\;e_{i}\rangle =\sum_{\ell =1}^{VT}x_{j,\;\ell }e_{i,\;\ell }$$

### 2.7. Kernel methods

Section 2.7.1 introduces *nonlinear similarity measures* by extending the dot product, a linear similarity measure introduced in section C.1, to a *nonlinear* setting by introducing *nonlinear* mappings, which are used by kPCA-*st* (introduced in section 2.7.2). Section 2.7.3 explains the pitfalls of applying kPCA in only the temporal or only the spatial dimensions.

#### 2.7.1. Nonlinear similarity measures

We followed Schölkopf and Smola (2002) to show that a *nonlinear* similarity measure can still exist in an inner product space. Using this approach, we tested whether the different values produced by using a nonlinear similarity measure (as opposed to a linear similarity measure) would improve the discrimination of patients from controls.

In order to use the inner product as a measure of similarity, the original data points x∈X were mapped into vectors in an inner product space ℋ:

(2) Φ:X→ℋ

Φ's embedding into ℋ allowed the introduction of different similarity measures that can be expressed as inner products:

(3) $$k_{\Phi }(x_{i},\;x_{j}):={\langle x_{i},\;x_{j}\rangle }_{\Phi }=\langle \Phi (x_{i}),\;\Phi (x_{j})\rangle$$

Mapping subject images to an inner product space through Φ(·) allowed us to investigate whether the enriched geometric relationship afforded by the mapping Φ(·) could improve the discriminatory power of a classifier.

For some Φ, one can compute *K*_Φ_(*x*_*i*_, *x*_*j*_) (Equation 2) directly, without first computing Φ(*x*_*i*_) and Φ(*x*_*j*_), using the “kernel trick.” The kernel trick allows one to *efficiently* compute the kernel function by avoiding explicit mapping of the data points *x*_*i*_ and *x*_*j*_ to the higher-dimensional Φ(*x*_*i*_) and Φ(*x*_*j*_). That is, using the kernel trick, one can compute each kernel value *k*(*x*_*i*_, *x*_*j*_) in a way that depends on the dimensionality of the original data points *x*_*i*_ and *x*_*j*_, rather than the potentially much higher dimensionality of Φ(*x*_*i*_) and Φ(*x*_*j*_). This approach makes it feasible to use nonlinear transforms which would be difficult, if not impossible, to represent explicitly (Herbrich, 2001). The *Radial Basis Function* (RBF) kernel is an example of this. For the RBF kernel, the range of the Φ(*x*) mapping is infinite dimensional. The RBF kernel value of two data points *x*_*i*_ and *x*_*j*_, in our original space, is

(4) $${\tilde{K}}_{\text{RBF}}(i,\;j)=k_{\text{RBF}}(x_{i},\;x_{j})=\exp\left(\frac{-\langle (x_{i}-x_{j}),\;(x_{i}-x_{j})\rangle }{2\sigma^{2}}\right)$$

where *x*_*i*_ and *x*_*j*_ represent two subjects' voxel waveforms as vectors and σ is a user-determined parameter. Note that the matrix containing the kernel values between *all* pairs of the data points is denoted as $\tilde{K}$. For ${\tilde{K}}_{\text{RBF}}$, the RBF subscript denotes the use of the RBF mapping.

When performing kPCA-*st* (section 2.7.2), we used the RBF kernel because the kernel matrix ˜**K**_RBF_ is strictly positive definite, which guarantees the recovery of *N* strictly positive eigenvalues. Therefore we always had *N* principal components to capture the variance in the data. Having fewer than *N* principal components could have hurt the diagnostic accuracy results, as there would have been fewer eigenvectors to capture the variance in the data.

#### 2.7.2. Kernelized PCA compression over space and time: kPCA-*st*

kPCA-*st* consisted of four basic steps:

1. Compute kernel matrix ${\tilde{K}}_{\text{RBF}}\in {\mathbb{R}}^{N\times N}$ over the training data (Equation 4).
2. Center ${\tilde{K}}_{\text{RBF}}$ using $K_{\text{RBF}}={\tilde{K}}_{\text{RBF}}-1_{N}{\tilde{K}}_{\text{RBF}}-{\tilde{K}}_{\text{RBF}}1_{N}+1_{N}{\tilde{K}}_{\text{RBF}}1_{N}$, where every element of the *N* × *N* matrix **1**_*N*_ is $\frac{1}{N}$.
3. Compute the eigenvector matrix **E**_RBF_ of **K**_RBF_.
4. Project **K**_RBF_ onto the eigenvector matrix (Equation A.8), where **X**_*st*_ and **E** were replaced with **K**_RBF_ and **E**_RBF_, respectively.

Figure 5 illustrates the process for kPCA-*st*, assuming PCA is replaced with kPCA. Similarly to PCA-*st*, kPCA-*st* produced *N* reduced imaging features for each subject.

We emphasize that every element in kPCA-*st*'s kernel matrix represents the point-wise similarity between two subjects' entire fMRI scans (after applying mapping Φ) because it measures the similarity of all T points for every voxel location.

Using kPCA-*st* as a dimensionality reduction process for fMRI is appealing because (1) the result is different from using the standard linear methods and therefore presents an alternative that might work more effectively and (2) the kernel matrix, **K**_RBF_, grows with the square of the number of data points instead of the dimensionality of the range of Φ. For all diagnostic tests using kPCA-*st*, we used the RBF kernel with the kernel parameter σ = 150.

#### 2.7.3. kPCA in only temporal dimension

Applying kPCA-*st* to fMRI data reduces dimensionality over *both* the spatial and temporal dimensions. We also considered applying kPCA in only the temporal dimension, as in PCA-*t*. However, this approach defeats the purpose of using kPCA over canonical PCA. Performing kPCA over the temporal dimension, using PCA-*t*'s data matrix **X**_*t*_, produces an *NV* × *NV* kernel matrix, which faces computational issues because of its large size. If we average over larger subvolumes to reduce the computational strain of calculating the kernel matrix, the loss of spatial information cripples performance. We considered averaging over 8 × 8 × 8 subvolumes (results not shown) but observed poor performance in comparison to averaging over 3 × 3 × 3 subvolumes. When averaging over 8 × 8 × 8 subvolumes, we believe that the large size of the kernel matrix and the poor result of kPCA-*st* are sufficient to dismiss applying kPCA in the temporal dimension.

If kPCA-*st* is applied to the *spatial* matrix—i.e., subject volumes at a single time point—then the data matrix **X** given as input to kPCA-*st* has dimensionality *N* × *V*. Given that there are *T* resting-state volumes per subject, it is difficult to justify selection of a specific time point's volume because all volumes were collected under the same conditions.

### 2.8. Testing methodology

We evaluated the dimensionality reduction methods in two settings. In two class diagnosis, we classified participants as ADHD (collapsed across subtypes) vs. healthy control. In three class diagnosis, we classified participants as ADHD combined (ADHD-c) type vs. ADHD inattentive (ADHD-i) type vs. healthy control. For each setting, we grouped the *seven* dimensionality reduction processes into *three* categories, depending on how each process transformed the averaged, BOLD signal intensity normalized, and masked original waveforms (see Figure 2). The number of processes in each category is parenthesized.

For the first category, we transformed each subject's waveforms as:

**I. Without FFT Transform** input to PCA-*t*, PCA-*st*, and kPCA-*st*. (3)

For the two remaining categories, we first computed the FFT for each subject's waveforms then used the magnitudes from each subject's FFTed waveforms as:

**II. FFT Only** the reduced-dimensionality imaging features. (1)
**III. With FFT Transform** input to the PCA variants. We differentiated these processes from those in the first category by referring to these variants as FFT + PCA-*t*, FFT + PCA-*st*, and FFT + kPCA-*st*. (3)

We used three feature sets for our evaluation:

**Phenotypic Data** consisted of subject age, gender, scanning site, and three IQ scores: Verbal, Performance, and Full IQ. Missing IQ values were set to the mean over all subjects who had the respective IQ score.
**Imaging Data** contained only the reduced-dimensionality imaging features produced by one of the seven dimensionality reduction processes.
**Imaging and Phenotypic Data** appended the phenotypic data to the imaging data returned by one of the seven dimensionality reduction processes^1^.

This gave a total of 15 feature sets for each setting, including the phenotypic only feature set as well as the “Imaging Data” and “Imaging and Phenotypic Data” feature sets for each of the seven dimensionality reduction processes.

We used each of the resulting feature sets as input to a linear kernel support vector machine (SVM) learner^2^, which produced a classifier. We then evaluated the feature sets in terms of the accuracy of the resulting classifier. This accuracy was based on 10-fold Cross Validation (Hastie et al., 2009), where we used the same folds for tests of different feature sets.

We compared every method's performance to the baseline accuracy derived by guessing the majority class (healthy control) for all participants. We also compared kPCA-*st*'s results to PCA-*t's.* It was our hypothesis that using kPCA-*st* would result in superior diagnostic accuracy compared to using canonical PCA for dimensionality reduction. To further test the results—i.e., to make sure they were not a consequence of overfitting—we used a holdout set to evaluate the performance of each method. At the close of the ADHD-200 Global Competition, the most accurate of the submitted classifiers was based on only the phenotypic data (Brown et al., 2012; Fair and Milham). Therefore, we also compared our results to automated diagnosis using a linear SVM with only phenotypic data as input.

## 3. Results

**Figure 6:**
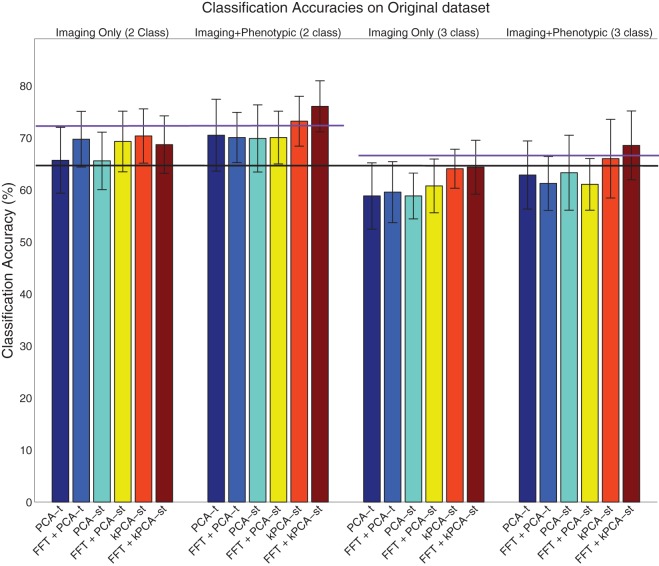
**Visualization of the results on the original dataset (Table 2), where the error bar is ± the standard deviation**. The purple lines denote the phenotypic data classification accuracy in the two class (left) and three class (right) setting, and the black line denotes the baseline in both the two and three class settings.

**Figure 7:**
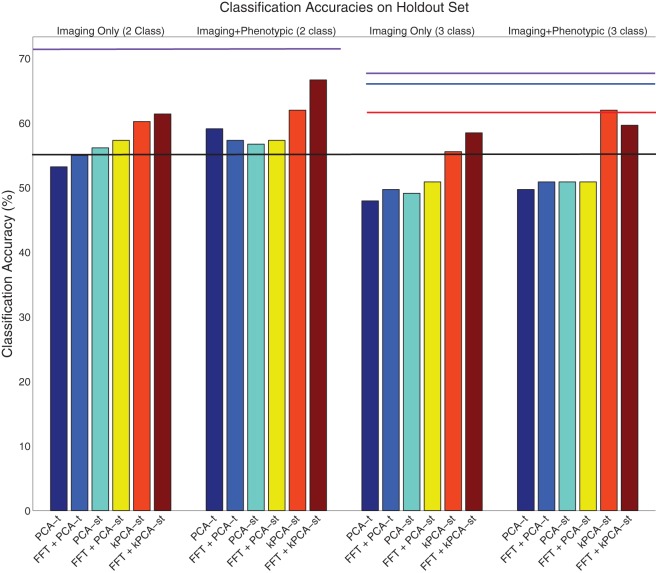
**Visualization of the results on the holdout dataset (Table 3)**. The purple lines denote the phenotypic data classification accuracy in the two class (left) and three class (right) settings. The black line denotes the baseline in both the two and three class settings. The red line denotes the accuracy of the ADHD-200 global competition official winners' submission. The blue line denotes the accuracy of our competition submission using the logistic classifier with phenotypic data only.

We discuss the performance of all dimensionality reduction methods on the original dataset, followed by the holdout set (sections 3.1 and 3.2). For both the original and holdout datasets (see Table 1), we initially discuss the performance of the PCA variants without using FFT (sections 3.1.1 and 3.2.1). We then discuss the performance achieved using FFT features (sections 3.1.2 and 3.2.2). We conclude each dataset's results subsection by discussing the performance using the FFT component weights as input to the PCA variants (sections 3.1.2 and 3.2.2). For all of these cases, we first discuss the results in the two class setting before the three class setting. In each setting, we first consider using only the imaging features without the phenotypic data and then consider the combination of the imaging features with the phenotypic data. Tables 2, 3 contain the results, shown in Figures 6, 7, for the original and holdout datasets, respectively. In section 3.3, we discuss control tests using different preprocessing of the fMRI data.

**Table 2:** **Two and three class classification accuracies on the *original 668 subject dataset***.

**Number of classes**	**Baseline**	**Phenotypic only**	**FFTed waveforms**	**PCA variant**	**Imaging only**	**Imaging + phenotypic data**
2	64.22	72.9	−	PCA-*t*	65.69 (7.16)	**70.51** (6.91)
			−	PCA-*st*	65.57 (5.51)	**69.89** (6.46)
			−	kPCA-*st*	70.35^*^(5.21)	**73.20** (4.79)
			+	−	**68.41** (5.50)	**70.95** (7.66)
			+	PCA-*t*	**69.60** (5.36)	**70.06** (4.83)
			+	PCA-*st*	**69.30** (5.82)	**70.06** (5.08)
			+	kPCA-*st*	**68.70** (5.53)	76.04^*^ (4.92)
3	64.22	66.77	−	PCA-*t*	58.82 (6.26)	62.86 (6.55)
			−	PCA-*st*	59.82 (6.16)	63.30 (6.55)
			−	kPCA-*st*	64.06^*^ (3.74)	66.0^*^ (7.56)
			+	−	63.92^*^ (5.88)	64.06 (4.37)
			+	PCA-*t*	59.56 (5.87)	61.23 (5.19)
			+	PCA-*st*	60.76 (5.17)	61.07 (4.97)
			+	kPCA-*st*	64.36^*^(5.19)	68.55^*^(6.61)

The reduced features were obtained by performing FFT and/or PCA-t, PCA-st, and kPCA-st on the imaging data. Standard deviations are provided in parenthesis. An accuracy value is in **bold** if it is statistically better than baseline, and it is asterisked (

* ) if it is statistically better than PCA-t in the same setting.

**Table 3:** **Two and three class classification accuracies on the *holdout set*, where the reduced features were obtained by performing FFT and/or PCA-*t*, PCA-*st*, and kPCA-*st* on the averaged imaging data**.

**Number of classes**	**Baseline**	**Phenotypic only**	**FFTed waveforms**	**PCA variant**	**Imaging only**	**Imaging + phenotypic data**
2	54.97	71.35	−	PCA-*t*	53.22	59.1
			−	PCA-*st*	56.14	56.73
			−	kPCA-*st*	60.23	61.99
			+	−	56.73	56.73
			+	PCA-*t*	54.97	57.31
			+	PCA-*st*	57.31	57.31
			+	kPCA-*st*	61.40	66.67
3	54.97	67.25	−	PCA-*t*	47.95	49.71
			−	PCA-*st*	49.12	50.88
			−	kPCA-*st*	55.56	61.99
			+	−	50.88	53.22
			+	PCA-*t*	49.71	50.88
			+	PCA-*st*	50.88	50.88
			+	kPCA-*st*	58.48	59.65

### 3.1. Original dataset results

#### 3.1.1. Without FFT transform

For each subject, PCA-*t* computed 370 component weights at each of 3584 voxel locations. We only used the *m* = 2 largest components (from each voxel) because they captured over 99.9% of the variance in the data. Both PCA-*st* and kPCA-*st* computed a total of 668 component weights for each subject. For PCA-*st*^3^ and kPCA-*st*, we used *m* = 667 and *m* = 668 components, respectively.

When using only the reduced imaging features from PCA-*t* or PCA-*st* in the two class setting, the accuracies were not statistically better than the baseline. In contrast, using only the reduced imaging features from kPCA-*st* produced an accuracy of 70.3%, which was statistically better than the baseline, PCA-*t*, and PCA-*st* (*p* = 0.0024, *p* = 0.014, and *p* = 0.022), but it was not statistically better than the accuracy achieved using only the phenotypic data.

When combining the imaging and phenotypic features in the two class setting, each of PCA-*t*, PCA-*st*, and kPCA-*st* produced classification accuracies that were statistically better than the baseline (*p* = 0.013, *p* = 0.016, and *p* = 0.00032). Even though PCA-*t*'s and PCA-*st*, when combined with phenotypic data, produced classification accuracies statistically better than the baseline, neither surpassed the phenotypic classification accuracy. That is, in the two class setting, using only the phenotypic data was better than combining it with the reduced imaging features from PCA-*st* and PCA-*t*. In contrast, kPCA-*st* combined with phenotypic data produced a superior, but not statistically better (*p* = 0.88), accuracy than using only the phenotypic data. In general for the two class setting, the discriminatory power of the classifier based on the kPCA-*st* derived imaging features was better than that of classifiers which used features from PCA-*t* or PCA-*st*.

In the three class setting, none of PCA-*t*, PCA-*st*, or kPCA-*st* produced classification accuracies that were statistically better than the baseline, using either the combination of imaging and phenotypic data or only the imaging data. While all of the feature extraction methods performed below the baseline when using only the imaging data in the three class setting, kPCA-*st* performed statistically better than PCA-*t* and PCA-*st* (*p* = 0.018 and *p* = 0.018). When combining the imaging and phenotypic data in the three class setting, kPCA-*st* produced accuracies that were not statistically better than baseline (*p* = 0.38), PCA-*t* (*p* = 0.063), or PCA-*st* (*p* = 0.10).

#### 3.1.2. Using FFT only

In both the two and the three class settings, the FFT was used to reduce the temporal dimensionality of the imaging data. In the two class setting when using the 633,040 imaging features or combining them with the phenotypic data, FFT produced accuracies that were statistically better than the baseline (*p* = 0.00055 and *p* = 0.0021). However, in the three class setting, FFT failed to produce accuracies equal to or above the baseline.

We show that FFT is more effective as a preprocessing step in the next subsection, based on the results from the FFT + PCA-*t*, FFT + PCA-*st*, and FFT + kPCA-*st* feature extraction methods.

#### 3.1.3. With FFT transform

For the tests described in this subsection, instead of applying PCA-*t*, PCA-*st*, and kPCA-*st* to the waveforms directly, we first applied the FFT as a *preprocessing step*. The goal was to test whether the magnitude of each FFT-derived frequency component was more diagnostically useful than the raw BOLD signal intensities. FFT transformed the imaging data's temporal dimensionality from 370 time points to 185 frequency component magnitudes. If we let *T* equal the number of frequency components, then the feature extraction methods outlined in Figures 4, 5 still apply.

FFT + PCA-*t* computed 185 PCA component weights at each of 3584 voxel locations for each subject. We only used the *m* = 2 largest PCA components from each voxel because they captured over 99.9% of the variance in the data. Both FFT + PCA-*st* and FFT + kPCA-*st* computed 668 component weights for each participant. For both FFT + PCA-*st* and FFT + kPCA-*st*, we used *m* = 668 components weights.

In the two class setting using only the imaging data, FFT + PCA-*t*, FFT + PCA-*st*, and FFT + kPCA-*st* each produced classification accuracies that were statistically better than the baseline (*p* = 0.0011, *p* = 0.0071, and *p* = 0.049). Combining FFT + PCA-*t* or FFT + PCA-*st*'s reduced imaging features with the phenotypic data resulted in classification accuracies that were statistically better than the baseline (*p* = 0.0011 and *p* = 0.0014). However, this performance was nearly identical to that using only FFT + PCA-*t* and FFT + PCA-*st*'s reduced imaging features. In other words, combining the phenotypic data with the features produced by FFT + PCA-*t* and FFT + PCA-*st* did not improve the discriminatory power of the classifier. Combining the phenotypic data with the reduced imaging features of FFT + kPCA-*st* resulted in a classification accuracy that was statistically better than the baseline, PCA-*t*, PCA-*st*, *and* the phenotypic data (*p* = 1.1 × 10^−7^, *p* = 0.0048, *p* = 0.0056, and *p* = 0.047). That is, using the FFT + kPCA-*st* imaging features with the phenotypic data improved the discriminatory power of the classifier.

In the three class setting, FFT + PCA-*t* and FFT + PCA-*st* performed similarly to the case where FFT was not used as a preprocessing step. That is, these approaches failed to produce classification accuracies that were statistically better than the baseline, whether or not they were combined with phenotypic data. FFT + kPCA-*st* failed to outperform the baseline when using imaging data only. FFT + kPCA-*st* produced a classification accuracy that was statistically better than FFT + PCA-*t* (*p* = 0.032) but not better than FFT + PCA-*st*'s accuracy. Combining the phenotypic data with FFT + kPCA-*st* imaging features produced a classification accuracy that was statistically better than the baseline (*p* = 0.0047), but it was not statistically better than the accuracy achieved using only the phenotypic data.

In summary, when using phenotypic and imaging data in the two class setting, classification based on the combination of FFT + kPCA-*st* features and phenotypic data performed better than classification based only on the phenotypic data. FFT + kPCA-*st* was the only method that produced a statistically better result than the phenotypic data in either the two or three class settings.

The results suggest that taking the magnitudes of all voxel waveforms' frequency components returned by FFT could be a useful *preprocessing* step for voxel waveforms.

### 3.2. Holdout set performance

The ADHD-200 Global Competition used a holdout set to evaluate the submissions. The organizers released this data after the competition concluded. We used 171 of the 197 subjects in the holdout set, where we excluded 26 subjects from a hospital that did not authorize the release of their diagnoses. For both the two and three class settings, the baseline accuracy on this dataset was 54.97%, derived by guessing “healthy control” for all subjects.

#### 3.2.1. Without FFT transform

In the two class setting, using only the reduced imaging features, PCA-*t*, PCA-*st*, and kPCA-*st* produced classification accuracies of 53.22%, 56.14%, and 60.23%. PCA-*t*'s accuracy was below the baseline. Using phenotypic data only resulted in a classification accuracy of 71.35%. These results are consistent with the cross-validation results from the original dataset. The phenotypic data outperformed any method that used only the reduced imaging data, and kPCA-*st* outperformed both PCA-*t* and PCA-*st*. When combining the phenotypic data with the imaging data in the two class setting, PCA-*t*, PCA-*st*, and kPCA-*st* performed better than the baseline but not better than the phenotypic data-based approach.

In the three class setting, both PCA-*t* and PCA-*st* failed to produce classification accuracies above the baseline. In contrast, the classification accuracy for kPCA-*st* using only the reduced imaging features was 55.56%, which was slightly better than the baseline. When the kPCA-*st* reduced imaging features were combined with the phenotypic data, a classification accuracy of 61.99% was produced.

#### 3.2.2. Using FFT only

Similarly to what was done with the original dataset, we used FFT as a preprocessing step, which provided the voxel waveform frequency component magnitudes as input to PCA-*t*, PCA-*st*, and kPCA-*st*. Interestingly, FFT's performance on the holdout dataset was not consistent with the results from the original dataset (see section 3.1.2). FFT failed to produce accuracies well-above baseline in either the two or three class settings with the holdout data. As we show in the next subsection, this preprocessing step improved the accuracy of each method.

#### 3.2.3. With FFT transform

In the two class setting using only the imaging data, FFT + PCA-*t* performed similarly to the baseline. FFT + PCA-*st* and FFT + kPCA-*st* outperformed the baseline by different margins, FFT + kPCA-*st*'s margin being larger than FFT + PCA-*st*'s. When combined with the phenotypic data, FFT + PCA-*t*, FFT + PCA-*st*, and FFT + kPCA-*st* all outperformed the baseline. FFT + kPCA-*st*'s performance was superior to those of FFT + PCA-*t* and FFT + PCA-*st*. Combining the phenotypic data with FFT + kPCA-*st*'s reduced imaging features failed to outperform the phenotypic data in the two class setting.

In the three class setting when using only the imaging data, FFT + PCA-*t* and FFT + PCA-*st* failed to outperform the baseline. In comparison, FFT + kPCA-*st* produced an accuracy of 58.48%, which was better than the baseline. When combining the phenotypic data with the imaging features, both FFT + PCA-*t* and FFT + PCA-*st* failed to outperform the baseline. FFT + kPCA-*st* produced an accuracy of 59.65%, which was better than the baseline but slightly worse than the 61.99% achieved using kPCA-*st* in the same setting.

Using FFT as a preprocessing step improved most methods' classification accuracies. kPCA-*st* still produced the best result of any method using imaging features and consistently outperformed PCA-*st* and PCA-*t*. In the two class setting, FFT + kPCA-*st* benefited the most from using FFT as a preprocessing step, as it produced classification accuracies better than the baseline *and* kPCA-*st* when using only the imaging data.

The results suggest that using FFT as a preprocessing step before applying dimensionality reduction can improve diagnostic accuracy in this context. Results from both the original dataset and the holdout set supported our hypothesis that reducing dimensionality over both the spatial and temporal dimensions, as kPCA-*st* does, produces better diagnostic accuracies than reducing only the temporal dimensionality, as PCA-*t* does.

### 3.3. Control tests with different preprocessing

Resting state fMRI scan durations and sampling rates were different for different data collection sites. In preprocessing the data, subject's fMRI scans were truncated to the shortest scan length in the dataset (185 s) and resampled to a 2 Hz sampling rate (see section 2.2). To investigate whether the truncation and re-sampling procedure may have had detrimental consequences on diagnostic accuracy, we tested diagnosis using the ADHD-200 Competition's preprocessed data. Given that images from different sites differed in their temporal dimensions, we only tested performance from one site, the NeuroIMAGE site (Fair et al.). When we compared kPCA-*st*'s performance on the Competition's preprocessed data to the result that used our preprocessed data, we observed that kPCA-*st* produced similar results for each dataset. This suggests that the temporal truncation and resampling in our preprocessing did not substantially impact performance.

We also tested diagnosis using fMRI data resampled into a 2.0 s volume time (0.5 Hz sampling rate), instead of a 0.5 s volume time. We found that our diagnostic methods did not perform statistically better than the baseline in this case. Up-sampling to 0.5 s volume time improved performance. A previous study focusing on slice-timing correction—i.e.. temporal data interpolation—found that slice-timing effects can significantly impair fMRI results, and slice-timing correction methods, such as linear interpolation, can successfully compensate for these issues, which improves the robustness of the analysis (Sladky et al., 2011). For this dataset, linear interpolation allows preservation of the temporal data for scans with higher sampling rates by introducing data points for scans with lower sampling rates. Linear interpolation is used to both preserve the voxels' activations and ensure that these activations “line up,” which is similar to how it is used in slice-timing correction. We therefore argue that up-sampling the fMRI data to a 2 Hz sampling rate is warranted.

## 4. Discussion

Our results suggest that applying kPCA (kPCA-*st*) leads to classifiers that are statistically better than canonical PCA (PCA-*t*) when using the imaging data only, in the context of imaging-based diagnosis of ADHD. In the two class setting, kPCA-*st* produced a classification accuracy that was statistically better than the baseline. The performance of kPCA-*st* in the two class setting provides a *proof-of-concept* as we work toward fMRI-based diagnostic protocols for use in the clinic.

Without using FFT to preprocess voxel waveforms, kPCA-*st* performed better than canonical PCA (PCA-*t*) when only using imaging data in both the two and three class settings. It also performed better than PCA-*t* in the three class setting when using both imaging and phenotypic data.

When FFT was used as a preprocessing step, FFT + kPCA-*st* performed better than FFT + PCA-*st* and FFT + PCA-*t* when using the imaging and phenotypic data in the two and three class settings. It also performed better than PCA-*t*, but not PCA-*st*, when using only the imaging data in the three class setting.

kPCA-*st*'s better performance over PCA-*t* in either the two or three class settings, without the FFT transform, suggests that reducing over spatial and temporal dimensions is superior to reducing over temporal dimensions, in this context. However, FFT + kPCA-*st* failed to produce a statistically better result than FFT + PCA-*t*, which suggests that using the FFTed waveforms greatly benefits PCA-*t* and marginally impacts kPCA-*st*. A possible explanation for this result is that kPCA-*st*'s advantage over PCA-*st* and PCA-*t* is mitigated by introducing a preprocessing step that reduces the temporal dimensions of the imaging data.

The results show that nonlinear mapping of subjects' fMRI data to a high-dimensional inner product space, as kPCA-*st* does, can increase discriminatory power of a classifier when compared to methods that do not, such as PCA-*t* and FFT.

Interestingly, FFT + kPCA-*st* when combined with phenotypic data outperformed the phenotypic data-only approach in *both* the two and three class setting, though only the result in the two class setting was statistically better than the phenotypic data. We believe that FFT + kPCA-*st*'s statistically insignificant improvement over the phenotypic data in the three class setting is not a large issue, because the phenotypic data-based approach itself was not statistically better than the baseline.

There are two potential consequences of our results:

1. Our results suggest that combining imaging and phenotypic data can improve the discrimination of ADHD subtypes from healthy controls. Using FFT + kPCA-*st*'s features combined with phenotypic data produced diagnostic accuracies that were better than using only the FFT + kPCA-*st*'s features or only the phenotypic data. This finding is particularly important given how challenging automated diagnosis with the ADHD-200 dataset has proven to be. Among the ADHD-200 Global Competition submissions, no imaging-based diagnostic method out-performed a fairly simple approach using the logistic classifier with phenotypic data inputs (see Brown et al., 2012). The current study demonstrates a means of improving upon such phenotypic-only methods.
2. Imaging data provided diagnostic utility in the two class but not the three class setting. Five of our imaging-based diagnostic methods performed above chance baseline for two class diagnosis, but none did so for three class diagnosis.

To elaborate on the second statement, we note that three class diagnosis is intrinsically more difficult than two class diagnosis. In three class diagnosis, patients correctly diagnosed as patients but with the wrong subtype count as complete errors when computing accuracy scores. We expect that the neurobiological profiles of different ADHD subtypes should look more similar to each other than to that of healthy controls. This would contribute to the difficulty of discriminating ADHD subtypes as is required in three class diagnosis. In addition, the dataset we used for testing had 141 ADHD-c type subjects and 98 ADHD-i type subjects, in contrast to 429 healthy control subjects. Given that there are over three times as many images for control subjects than ADHD-c type patients, the second largest class, including additional ADHD patient scans may improve accuracy in the three class setting.

### 4.1. Challenges and limitations

Applying kPCA-*st* to fMRI data is in its infancy. This article focuses on its application to resting-state fMRI data. Future work should investigate its applicability to other settings such as task-based fMRI. In the context of fMRI-based diagnosis, it would be useful to investigate the impact of kernel and parameter selection on the learner's discriminatory power in future work.

#### 4.1.1. Differences in scanning protocols

The ADHD-200 data were collected from multiple hospitals across the world. One consequence with this large data release is that the MRI scanner hardware and scanning protocols differed among hospitals (Fair et al.). The resulting variability in the data may have obscured diagnostically-useful patterns in the imaging data.

#### 4.1.2. Temporal normalization of imaging data

**Figure 8:**
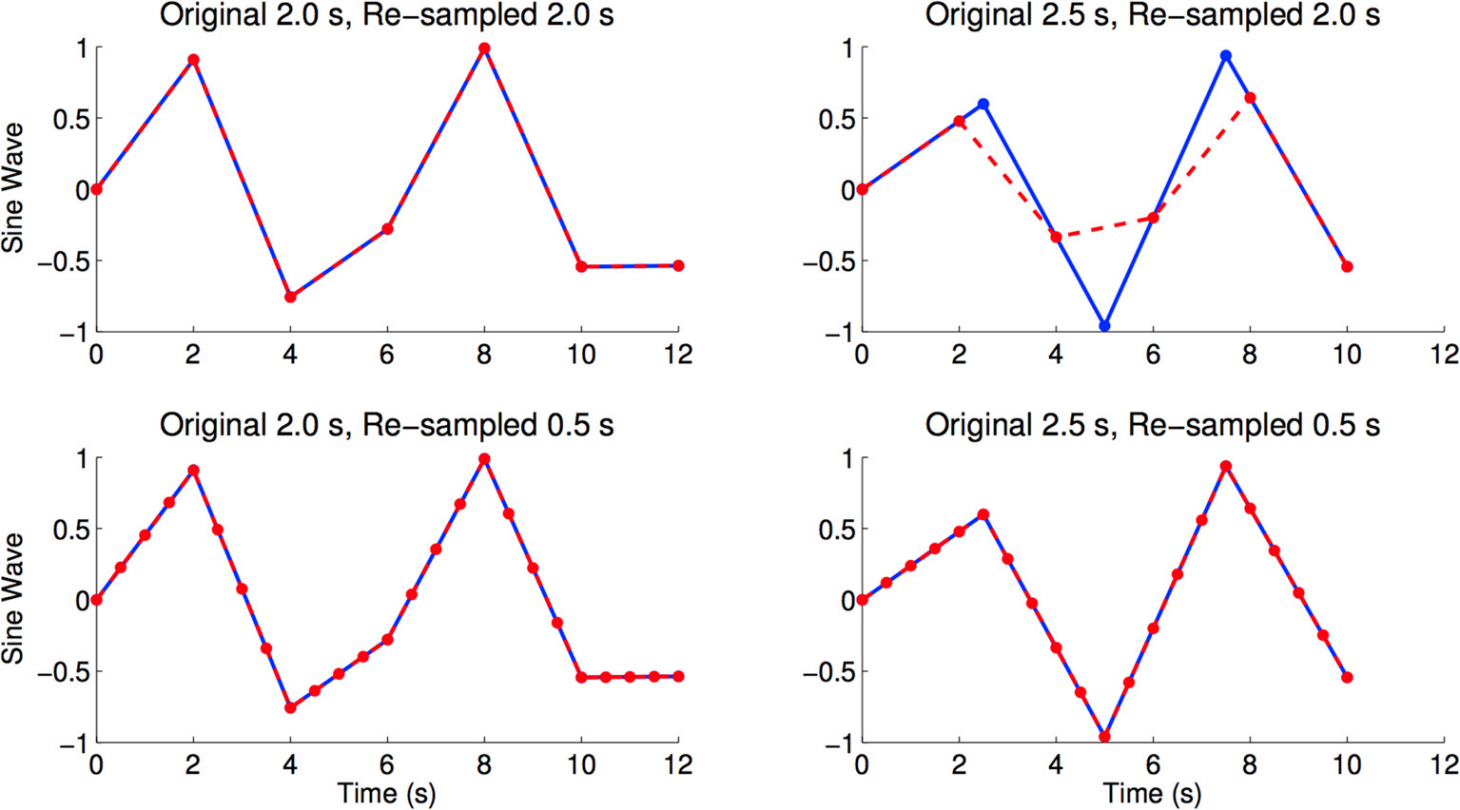
**Figures shows sine waves (blue) with sampling periods of 2.0 and 2.5 s, as well as re-sampled versions (red) of the same curves with sampling periods of 2.0 and 0.5 s**. The re-sampled curves were linearly interpolated from the blue curves. We see re-sampling errors caused by re-sampling from 2.5 to 2.0 s, where the maximum and minimum values of the red curve are very different from the blue curve. In the bottom right and bottom left panels we see that linear re-sampling does not have these errors, but acknowledge that the time points in-between originally sampled time points are extrapolated using linear interpolation (Brown et al., 2012).

The resting state fMRI scans from the ADHD-200 dataset were collected with different volume times, where the number of subjects are parenthesized: 1.5 s (88), 1.96 s (48), 2.0 s (410), 2.5 s (221), and 3.0 s (1). If we were to re-sample all scans into a 2.0 s volume time, there would be interpolation errors for those scans originally sampled at 1.5, 2.5, and 3.0 s (see Figure 8 for an illustration of this effect). To minimize temporal re-sampling errors, we therefore up-sampled all scans to a 0.5 s volume time (2 Hz sampling rate) using linear interpolation, as opposed to re-sampling into an intermediate volume time such as 2.0 s. Up-sampling into a 0.5 s volume time avoided introducing such re-sampling errors because the original volume times are integer multiples of 0.5 s^4^.

#### 4.1.3. *n*-fold cross validation prediction error

It is standard practice to use Cross Validation to estimate the expected prediction error. However, estimating the variance of the prediction error is not. Prediction error is a function of the training and test sets, where the training sets have some overlap and the test sets do not. The overlap of training sets leads to underestimation of the variance of the prediction error (Bengio and Grandvalet, 2004). We therefore acknowledge that the standard deviation quantities given for the classification accuracies in Table 2 could be underestimated, which *may* introduce bias when comparing these quantities from different dimensionality reduction processes.

#### 4.1.4. Masking

In section 2.4, we mentioned that a mask was used to exclude the waveforms of the voxels outside of the brain. Applying the mask *before* BOLD signal normalization dramatically impacted the classification accuracy, significantly decreasing the classification accuracy as a direct result of ignoring voxel locations outside of the brain prior to performing BOLD signal normalization. We observed that all voxels outside of the brain had negative values and all voxels inside the brain had positive values. The magnitude of the voxels outside of the brain depended on the scanning site, and applying the mask prior to BOLD signal normalization ignored information that we believe should be included. Thus, all images were masked *after* BOLD signal normalization was applied over the averaged image.

We hold that normalizing the signal for *all* 6688 spatial locations *prior* to applying the mask allowed the BOLD signal to be retain information about the site it was from. Future work should thoroughly investigate the impact of masking prior to BOLD signal normalization after performing image registration using fMRIs from different sites and/or scanners.

### 4.2. Summary

We have shown that combining phenotypic data with FFT + kPCA-*st* feature extraction applied to resting state fMRI data can produce automated diagnosis of ADHD that is more accurate that using only phenotypic data. This result builds on and improves upon previous phenotypic-only diagnostic approaches (Brown et al., 2012).

#### Conflict of interest statement

The authors declare that the research was conducted in the absence of any commercial or financial relationships that could be construed as a potential conflict of interest.

## Footnotes

^1^This feature set allowed us to explore whether the combination of the imaging and phenotypic features would have a *synergistic* effect.

^2^This is a standard learning algorithm. We considered other learners, but found none worked better.

^3^The smallest eigenvalue was zero for PCA-*st*.

^4^Note: 1.96 s is not an exact integer multiple of 0.5 s, but it is close. Due to memory constraints, it would not have been practical to use a re-sampled volume time of 0.02 s, which is the largest integral divisor of all the original volume times.

## Appendix

### C. Principal component analysis

Section C.1 introduces *linear similarity measures*, which are used in section C.2 to illustrate the relevant theoretical properties of PCA.

#### C.1. Linear similarity measures

We want the learners to produce classifiers that can *generalize* to unseen data points. Assuming that there are *c* classes in the data, when a new data point *x*_*N* + 1_ is provided, the classifier will assign *x*_*N* + 1_ a label belonging to one of these *c* classes. The class assigned by the learner for data point *x*_*N* + 1_ should contain data points similar to *x*_*N* + 1_, based on some notion of *similarity*. Before defining such a similarity measure, Schölkopf and Smola (2002) formalize the problem setting as follows.

Let X be the nonempty set containing the empirical data, and {1, …, *c*} be the set containing the class labels. Here the *i*th data point with the associated label is $\left(x_{i},\;y_{i})\in X\times \{1,\;\ldots ,\;c\right\}$ for *i* = 1, …, *N*. We assume that $X={\mathbb{R}}^{p}$ – i.e., data points are *p*-dimensional. In general, we use

(A.1) k:X×X→ℝ

for some similarity function *k* that outputs a *real value* that characterizes the similarity between a pair of data points. Here, we can use the *dot (inner) product*, also called the linear kernel, as a similarity function

(A.2) $$k_{\text{linear}}(x_{i},\;x_{j})=\langle x_{i},\;x_{j}\rangle =\sum_{\ell =1}^{p}x_{i,\ell }x_{j,\ell }$$

where *x*_*i*_ = [*x*_*i*, 1_,…, *x*_*i, p*_] and *x*_*j*_ = [*x*_*j*, 1_, …, *x*_*j, p*_] are data points in ℝ^*p*^.

Interpreted geometrically, the inner product between two data points *x*_*i*_ and *x*_*j*_ is the cosine of the angle, assuming that both data points are normalized to length 1, where the length, or norm, of a data point *x* is defined as $||x|{|}_{2}=\sqrt{\langle x,\;x\rangle }$. Note that this dot product is largest when *x*_*i*_ = *x*_*j*_ (i.e., are extremely similar) and is zero when *x*_*i*_ is orthogonal to *x*_*j*_.

A related similarity measure is correlation; similarity measures are a very general mathematical concept (Herbrich, 2001).

#### C.2. Theory of PCA

We introduce the theory of PCA in a general form, presenting information that is used in sections 2.6.1, 2.6.2, and 2.7.2. Let **X** be the *N* × p matrix, where each row corresponds to an observation and each column is centered around its mean. We can define a matrix of similarities

(A.3) $$\begin{array}{l} \;K=XX^{T}\\ K(i,\;j)=k_{\text{linear}}(x_{i},\;x_{j})=\langle x_{i},\;x_{j}\rangle =\sum_{\ell =1}^{p}x_{i,\ell }x_{j,\ell } \end{array}$$

where *x*_*i*_ and *x*_*j*_ are rows of **X**. Then

(A.4) Ke=λe

holds for each eigenvalue/eigenvector pair (λ, *e*), where *eigenvector e* ∈ ℝ^*N*^ has the corresponding *eigenvalue* λ ∈ ℝ. Since **K** is a Gramian matrix, it will always have non-negative eigenvalues λ ≥ 0. Assume that the eigenvalues of **K** are sorted in descending order—i.e., λ_*i*_ ≥ λ_*i* + 1_. The eigenvectors of a symmetric matrix are mutually *orthogonal* (Khuri, 2003). Note there are at most *N* non-zero eigenvalues with the corresponding eigenvector matrix **E**_*N*_ = [e_1_, …, e_*N*_] ∈ ℝ^*N* × *N*^.

Notice that if there many more observations than features—i.e., *N* » *p*—then **K** can be very large. The *dual* of PCA (or the dual trick) provides an easy way to compute the eigenvalue/eigenvector pairs of such a high-dimensional similarity matrix, by using the eigenvalue/eigenvector pairs of the lower-dimensional covariance matrix $S=\frac{1}{p-1}X^{T}X\in {\mathbb{R}}^{p\times p}$ (Jolliffe, 2002):

(A.5) Se=λe

(A.6) $$\begin{array}{l} X(X^{T}X)e=X\lambda e\\ \;K(Xe)=\lambda (Xe) \end{array}$$

which proves that the eigenvalue/eigenvector pairs (λ, **e**), computed using Equation (A.5), of the relatively-small *p* × *p* matrix **S**, correspond to the eigenvalue/eigenvector pairs (λ, **Xe**) of the much larger *N* × *N* similarity matrix **K**. That is, each (λ, **e**) eigenvalue/eigenvector pair of **S** corresponds to the eigenvalue/eigenvector pair of **K**: (λ, **Xe**). As *N* » *p*, it follows from Equation (A.4) that there are at most *p* eigenvalue/eigenvector pairs with corresponding eigenvector matrix **E**_*p*_ = [**e**_*1*_, …, **e**_*p*_] ∈ ℝ^*N* × *p*^.

Note that **E**_*p*_ can also be viewed the matrix that uses the *low-dimensional* eigenvector matrix **E**_*S*_ = [**e**_*1*_,…,**e**_*p*_] ∈ ℝ^*p* × *p*^ of **S** to redefine the coordinate system of **X**, which produces the *principal component score matrix*

(A.7) $$Z_{S}=XE_{S}\in {\mathbb{R}}^{N\times p}$$

where rows of **Z**_*S*_ are the data points in the new coordinate system, and columns are the *scores* of the *N* data points, where the *i*th column contains the *N* scores from the *i*th eigenvector of **S**.

Although Equation (A.7) shows that the score matrix is *numerically* equivalent to **E**_*p*_, *its interpretation depends on the application*: When we use the dual trick, we view **E**_*p*_ as the eigenvector matrix that contains *p N*-dimensional eigenvectors that allow us to *project* the dataset onto these eigenvectors, which produces the principal component matrix

(A.8) $$Z=E_{p}^{T}X\in {\mathbb{R}}^{p\times p}$$

where we omit this step when interpreting **E**_*p*_ as the score matrix.

The *i*th principal component is viewed as the data matrix *projected* onto (that is, multiplied by) the eigenvector **e**_*i*_. If there are a total of *N* principal components, the proportion of variance captured in the dataset by the first (largest) *m* < *N* principal components is given by:

(A.9) $$\text{proportion of variance}(\lambda_{\text{1}},\;\ldots ,\;\lambda_{\text{m}})=\frac{\sum_{i=1}^{m}\lambda_{i}}{\sum_{j=1}^{N}\lambda_{j}}$$

We select the *m* for each dimensionality reduction process as the number of components needed to capture over 99% of the variance.
